# Evaluation von Mehrwegtextilien im Operationssaal unter dem Aspekt der Nachhaltigkeit

**DOI:** 10.1007/s00113-026-01740-9

**Published:** 2026-07-28

**Authors:** Annika Steinmeier, Nicole Feder, Robert Mahlow, Ulrich Stöckle, Oliver Birkelbach, Robert Zahn

**Affiliations:** 1 Centrum für Muskuloskeletale Chirurgie, Charité – Universitätsmedizin Berlin, Augustenburger Platz 1, 13353 Berlin, Deutschland; 2 Klinik für Orthopädie, Universitätsklinikum Ulm, Oberer Eselsberg 45, 89081 Ulm, Deutschland

**Keywords:** Mehrwegabdeckungen, OP-Abdeckungen, Abfallmanagement, Krankenhausabfälle, Abfallreduktion, Reusable drapes, Surgical drapes, Waste management, Hospital waste, Waste reduction

## Abstract

**Hintergrund:**

Das Abfallmanagement im Krankenhaus stellt einen wesentlichen Faktor in Bezug auf ökologische Nachhaltigkeit dar. Abfälle aus Operationen müssen häufig unter hoher CO_2_-Emission vernichtet werden. Neben Recycling ist Abfallvermeidung eine wichtige Maßnahme zur Reduktion der CO_2_-Emissionen. Mehrwegtextilien stellen eine vielversprechende Möglichkeit zur Abfallreduktion in Operationssälen dar.

**Ziel der Arbeit:**

Evaluation von Mehrwegabdeckungen in Bezug auf anfallende Abfälle sowie auf Abläufe und Akzeptanz des Personals.

**Material und Methoden:**

In dieser prospektiven Beobachtungsstudie wurde der anfallende Abfall 272 orthopädisch-unfallchirurgischer Operationen gemessen. Je zwei Wochen erfolgte die Nutzung von Einwegabdeckungen sowie nachfolgend von Mehrwegabdeckungen. Es wurden Volumen sowie Gewicht des Abfalls sowie Vor‑, Nachbereitungs- und Operationszeit erfasst und statistisch ausgewertet. Im Anschluss erfolgte die Befragung des Personals zur Nutzung von Mehrwegtextilien.

**Ergebnisse und Diskussion:**

Während unter Nutzung von Einwegabdeckungen 93,59 (± 75,16) l bzw. 5,32 (± 2,04) kg krankenhausspezifischer Abfall pro Operation anfiel, konnte diese unter Nutzung von Mehrwegabdeckungen signifikant um 40 % des Volumens bzw. 34 % des Gewichtes gesenkt werden (*p* < 0,001). Es zeigte sich keine Differenz der Dauer der Vor- und Nachbereitung oder der Operation. Die dauerhafte Nutzung von Mehrwegtextilien im Operationssaal wurde von 96 % des befragten Personals als unproblematisch eingeschätzt.

Durch Nutzung von Mehrwegtextilien im Operationssaal kann der Abfall signifikant reduziert werden, ohne die Abläufe der Operationen zu beeinflussen; dies wird vom Personal befürwortet.

**Graphic abstract:**

**Figure:**
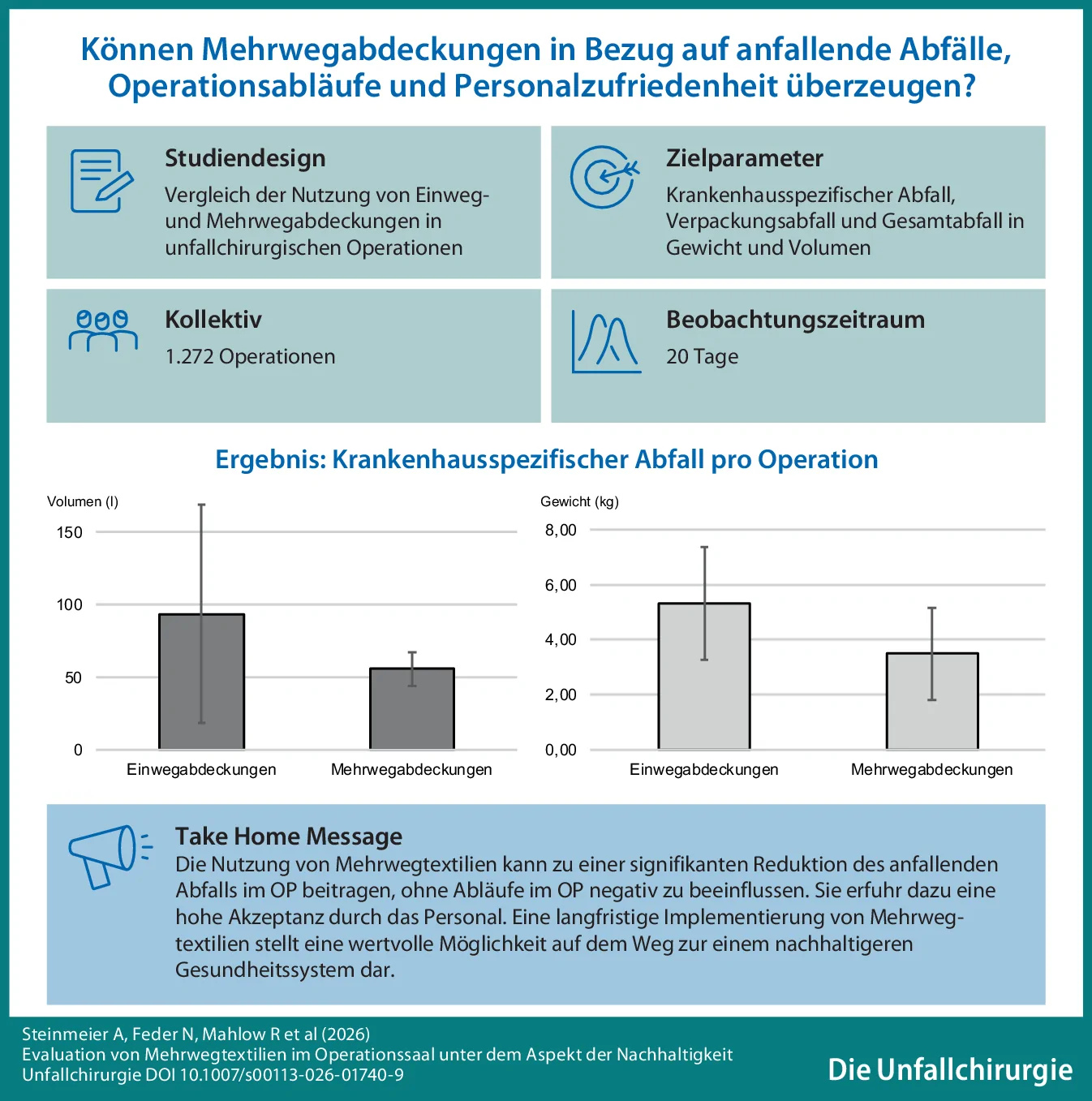

## Abstract (en)

**Background:**

Waste management in hospitals is an important factor with respect to environmental sustainability. Surgical waste disposal causes substantial CO_2_ emissions. In addition to recycling, waste reduction is an essential strategy for reducing emissions. Reusable textiles offer a promising approach to decrease waste in operating rooms.

**Objective:**

Assessment of reusable textiles in terms of waste generation, operational processes and staff acceptance.

**Material and methods:**

This prospective observational study compared waste generation when using disposable or reusable drapes in 272 orthopedic and trauma operations over periods of 2 weeks each. Waste volume and weight as well as preparation, clean-up and surgery times were recorded and statistically analyzed. Surgical staff were then asked about their experiences with reusable textiles.

**Results and discussion:**

The use of disposable drapes resulted in 93.59l (± 75.16l) and 5.32kg (± 2.04kg) of hospital-specific waste per operation. The introduction of reusable drapes reduced waste volume by 40% and weight by 34% (*p* < 0.001) without affecting preparation, clean-up or surgical time. Of the staff 96% considered the use of reusable textiles to be unproblematic. The use of reusable textiles significantly reduces waste in operating rooms without affecting workflows and is supported by operating room staff. The implementation represents a practical and effective measure towards greater sustainability in operating rooms.

## Hinführung zum Thema

Der Gesundheitssektor ist für einen relevanten Anteil der Umweltauswirkungen verantwortlich, die die globale Weltgesundheit bedrohen. Es ist dringend notwendig, das Gesundheitssystem nachhaltiger zu gestalten. Abfallmanagement ist ein wichtiger Aspekt in Bezug auf ökologische Nachhaltigkeit. Operationsabfälle werden häufig unter hoher Umweltbelastung vernichtet. Neben Abfalltrennung und Recycling stellt Abfallvermeidung eine wichtige Maßnahme zur Reduktion der Umweltschädigung dar. Mehrwegtextilien zeigten bereits vielversprechendes Potential zur Abfallreduktion in Operationssälen.

## Einleitung

Der Gesundheitssektor ist weltweit für ca. 4,4 % der Treibhausgasemission und bis zu 8,5 % der Umweltauswirkungen verantwortlich, und der Anteil steigt stetig weiter an [1, 2]. Folgen sind eine Zunahme von Extremwettern, Wassermangel sowie erhöhten Risiken für Infektions- sowie Herz- und Lungenerkrankungen [1, 3]. Daneben zeigt sich auch eine wirtschaftliche Relevanz, da neben steigenden Kosten für das Gesundheitswesen auch eine Einschränkung der Arbeits- und Erwerbstätigkeit prognostiziert wird [4]. Der Klimawandel wird daher bereits jetzt als größte globale Bedrohung der Weltgesundheit des 21. Jahrhunderts angesehen [5]. Es ist dringend notwendig, Maßnahmen gegen dieses Gesundheitsrisiko einzuleiten und auch das Gesundheitssystem selbst nachhaltiger zu gestalten.

Im Krankenhaus stellt das Abfallmanagement neben Energieaufwendung für Strom und Heizung einen wesentlichen Faktor dar. Pro Jahr entstehen ca. 5–6 Mio. Tonnen Abfall allein im deutschen Gesundheitssektor [6]. Damit liegt der täglich anfallende Abfall in Krankenhäusern mit ca. 6 kg/Patient:innen deutlich über dem in Privathaushalten mit 1,7 kg/Person [6]. Abfall aus dem Operationstrakt (OP) macht ca. 30 % des gesamten Krankenhausabfalls aus, und die Orthopädie und Unfallchirurgie produziert mehr Abfälle als jede andere Fachrichtung [7]. Das Ziel in Richtung eines nachhaltigeren Gesundheitssystems beschreibt dahingehend einen Plan zur Reduktion der Treibhausgasemissionen unter den zentralen Aspekten der Reduktion, des Recyclings und der Wiederverwertung, der Nutzung von erneuerbaren Energien sowie der Förderung von Forschung [8].

Durch effiziente Abfall- und Wertstofftrennung recyclebarer Materialien konnte bereits eine relevante Reduktion der krankenhausspezifischen Abfälle und damit der CO_2_-Emissionen nachgewiesen werden [9]. Daneben stellt die Abfallvermeidung eine wichtige Maßnahme zur Reduktion der CO_2_-Emissionen dar [8, 10]. Mehrwegprodukte können durch die Wiederverwendung zur Nachhaltigkeit im ökologischen und im ökonomischen Sinne beitragen, da der im OP entstehende krankenhausspezifische Abfall unter hoher CO_2_-Emission von 1–3 kg CO_2_-Emissionen/kg Abfall vernichtet werden muss [2].

Neben bereits etablierten Mehrweginstrumenten sind Mehrwegtextilien, wie sterile OP-Abdeckungen und OP-Kittel, eine vielversprechende Möglichkeit zu weiterer Abfallreduktion [11]. In bisherigen Studien konnte bereits eine gute Umweltbilanz in Bezug auf Energie- und Wasserverbrauch sowie CO_2_-Emissionen gezeigt werden [11].

Während Mehrwegtextilien über viele Jahrzehnte den Standard im OP darstellten, wurden sie in den vergangenen Jahrzehnten in vielen Kliniken, insbesondere aufgrund von Bedenken hinsichtlich hygienischer Sicherheit und vermeintlich logistischer Vorteile, durch Einwegtextilien ersetzt [12]. Mit der Weiterentwicklung moderner Mehrwegtextilien sowie zunehmenden ökologischen Anforderungen gewinnt deren erneute Bewertung jedoch zunehmend an Bedeutung. Wirtschaftswissenschaftliche Analysen zeigten zudem, dass Mehrwegsysteme gegenüber Einwegmaterialien wirtschaftlich konkurrenzfähig sind und sogar Einsparpotential bieten können [12, 13].

Ziel dieser prospektiven Studie war der direkte Vergleich von sterilen Einweg- und Mehrwegabdeckungen in Bezug auf den anfallenden Abfall und die daraus kalkulierten CO_2_-Emissionen. Außerdem sollten der Einfluss auf Vor- und Nachbereitungszeit sowie Operationsdauer ermittelt und die Zufriedenheit des Personals unter anderem in Bezug auf Komfort und Handling evaluiert werden.

Die Hypothese dieser Studie ist, dass der anfallende krankenhausspezifische Abfall sowie die daraus resultierenden CO_2_-Emissionen durch die Nutzung von Mehrwegabdeckungen signifikant reduziert werden können, ohne Nachteile in Bezug auf Handling oder Komfort zu haben.

## Material und Methoden

Die prospektive quasiexperimentelle Beobachtungsstudie im vergleichenden Querschnittdesign wurde über einen Zeitraum von vier Wochen in der unfallchirurgisch-orthopädischen Abteilung eines Maximalversorgers und Level-I-Traumazentrums einer deutschen Universitätsklinik durchgeführt. Dabei erfolgte die Erhebung des anfallenden Abfalls in fünf unfallchirurgischen OP. Eingeschlossen wurden alle unfallchirurgisch-orthopädischen Operationen des Tagesprogramms an Werktagen. Operationen, die im Rahmen der Notfallversorgung am Wochenende, an Feiertagen oder außerhalb des Regelbetriebs erfolgten, wurden ausgeschlossen.

Die Erhebung erfolgte zunächst über einen Zeitraum von zwei Wochen mit bereits etablierten regulär genutzten Mehrwegkitteln und Einwegabdeckungen. Nach ausführlicher interprofessioneller Schulung über Nutzen und Anwendung von Mehrwegabdeckungen erfolgte über einen Zeitraum von zwei Wochen die Erfassung des Abfalls bei Nutzung von Mehrwegkitteln und -abdeckungen. Die Anwendung, inklusive Verpackung und Fixierung der Abdeckungen mittels integrierter Klebestreifen, war bei Einweg- und Mehrwegmaterialien identisch, und die Logistik sowie die Aufbereitung der Mehrwegmaterialien erfolgten jeweils über den externen Dienstleister.

Für die Erhebung der Abfallmenge wurden das Volumen anhand der gefüllten Müllsäcke sowie das Gewicht separat für jede Operation von Beginn der Operationsvorbereitungen bis zum Abschluss der Nachbereitungen nach Verlassen der Patienten aus dem OP gemessen. Anfallender krankenhausspezifischer Abfall und potentiell recyclebare Wertstoffe wurden separat gemessen. Nicht aufgeführt wurden dabei Papierverpackungen und Kartonagen, die bereits vor Transport in den OP-Trakt in einer separaten Recycling-Tonne entsorgt wurden.

Außerdem wurde die Dauer der Vor- und Nachbereitung des OP-Funktionsdienstes jeder Operation in Minuten erfasst.

Im Anschluss an die Erhebungen erfolgte die Befragung des OP-Personals zur Evaluation der Mehrwegtextilien (Kittel und Abdeckungen) über anonymisierte Fragebögen über einen Zeitraum von 3 Tagen. Die Fragebögen erfassten demografische Daten, wie Alter und Berufserfahrung, sowie die konkrete Tätigkeit während der stattgehabten Operation. Auf einer fünfstufigen Skala wurden die subjektive Bedeutung von Nachhaltigkeit im Allgemeinen sowie im OP sowie die subjektive Abschätzung des Beitrags von Mehrwegtextilien zur Nachhaltigkeit und Materialkomfort, Handhabung sowie hygienische Sicherheit in Bezug auf Mehrwegmaterialien erfragt. Dazu wurden verschiedene Aspekte zwischen Einweg- und Mehrwegtextilien gegenübergestellt sowie Probleme im Umgang mit den Materialien und die subjektive Bedeutung zu einer langfristigen Etablierung erfasst.

Zur deskriptiven Statistik wurden bei intervallskalierten Daten Mittelwerte und Standardabweichungen ermittelt und Differenzen zwischen unabhängigen Variablen in Abhängigkeit der Datenverteilung mittels t‑Test bzw. Mann-Whitney-U-Test berechnet. Für nominalskalierte Variablen wurden absolute und relative Häufigkeiten angegeben und der Chi-Quadrat-Test zum Vergleich genutzt. Das Signifikanzniveau wurde auf 0,05 festgelegt. Zur statistischen Auswertung wurde die IBM SPSS Statistics Software (Version 29.0.0) angewendet.

## Ergebnisse

Unter Nutzung von Einwegabdeckungen fielen an 10 Tagen insgesamt 20970 l bzw. 889,36 kg Gesamtabfall bei 142 Operationen an, was 147,68 (± 79,42) l bzw. 6,26 (± 2,34) kg Abfall pro Operation entspricht. Unter Separation des potentiell recyclebaren Wertstoffes wurden im Mittel 93,59 (± 75,16) l bzw. 5,32 (± 2,04) kg krankenhausspezifische Abfälle gemessen. Diese machten damit ca. 63 % des Volumens bzw. ca. 85 % des Gewichtes des Gesamtabfalls aus.

Unter Nutzung von Mehrwegabdeckungen wurden an 10 Tagen 14250 l bzw. 593,69 kg Gesamtabfall bei insgesamt 130 Operationen gemessen, und es zeigte sich eine signifikant geringere Gesamtabfallmenge von 109,62 (± 19,66) l (*p* < 0,001) bzw. 4,57 (± 1,92) kg pro Operation (*p* < 0,001).

**Abb. 1:**
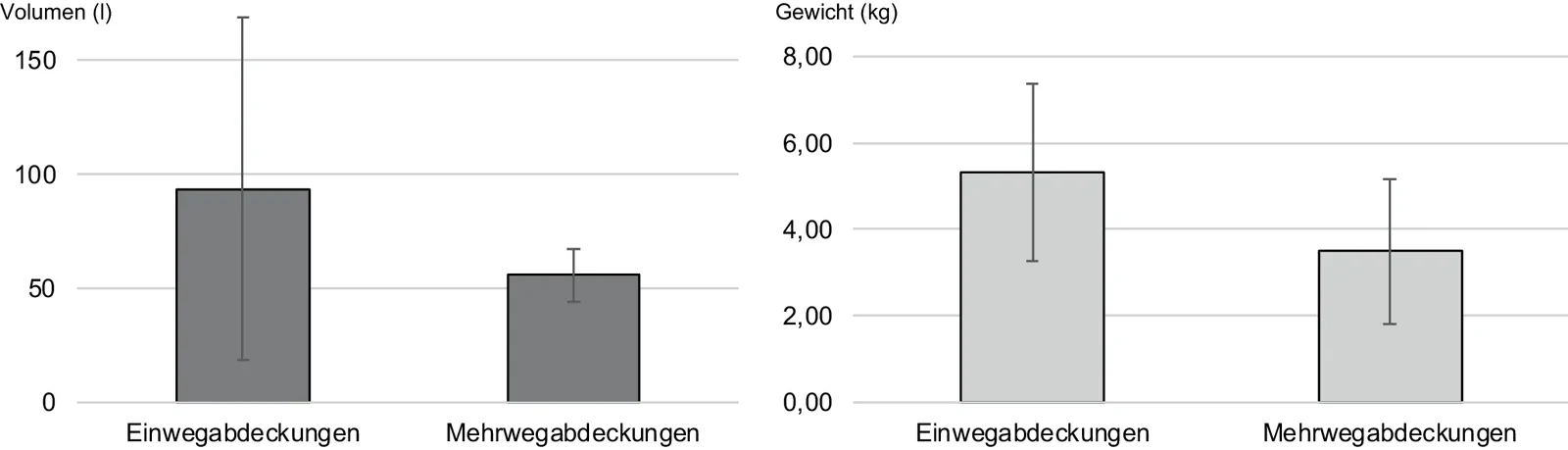
Reduktion von krankenhausspezifischem Abfall durch Nutzung von Mehrwegabdeckungen: Es zeigte sich durch die Nutzung von Mehrwegabdeckungen gegenüber Einwegabdeckungen eine signifikant geringere Menge an krankenhausspezifischem Abfall, sowohl in Bezug auf das Volumen als auch auf das Gewicht (*p* < 0,001)

Auch die krankenhausspezifischen Abfälle waren unter Nutzung von Mehrwegabdeckungen signifikant geringer mit 55,85 (± 11,67) l (*p* < 0,001) bzw. 3,5 (± 1,68) kg pro Operation (*p* < 0,001), was einer Reduktion um ca. 40 % des Volumens und 34 % des Gewichtes im Vergleich zur Nutzung von Einwegabdeckungen entsprach (Abb. 1). Bei potentiell recyclebaren Wertstoffen wurde dagegen ein signifikant höheres Gewicht pro Operation von 1,07 (± 0,50) kg unter Nutzung von Mehrwegabdeckungen gegenüber 0,95 (± 0,52) kg bei Nutzung von Einwegabdeckungen gemessen (*p* = 0,021), wobei das Volumen keine signifikanten Differenzen aufwies (*p* = 0,991).

Es zeigte sich im Vergleich zwischen Nutzung von Einweg- oder Mehrwegabdeckungen kein signifikanter Unterschied der Vor- und Nachbereitungszeiten (57,29 (± 23,09) min vs. 57,65 (± 23,46) min; *p* = 0,82) oder der Operationsdauer (83,49 (± 53,49) min vs. 75,11 (± 45,37) min; *p* = 0,25).

**Abb. 2:**
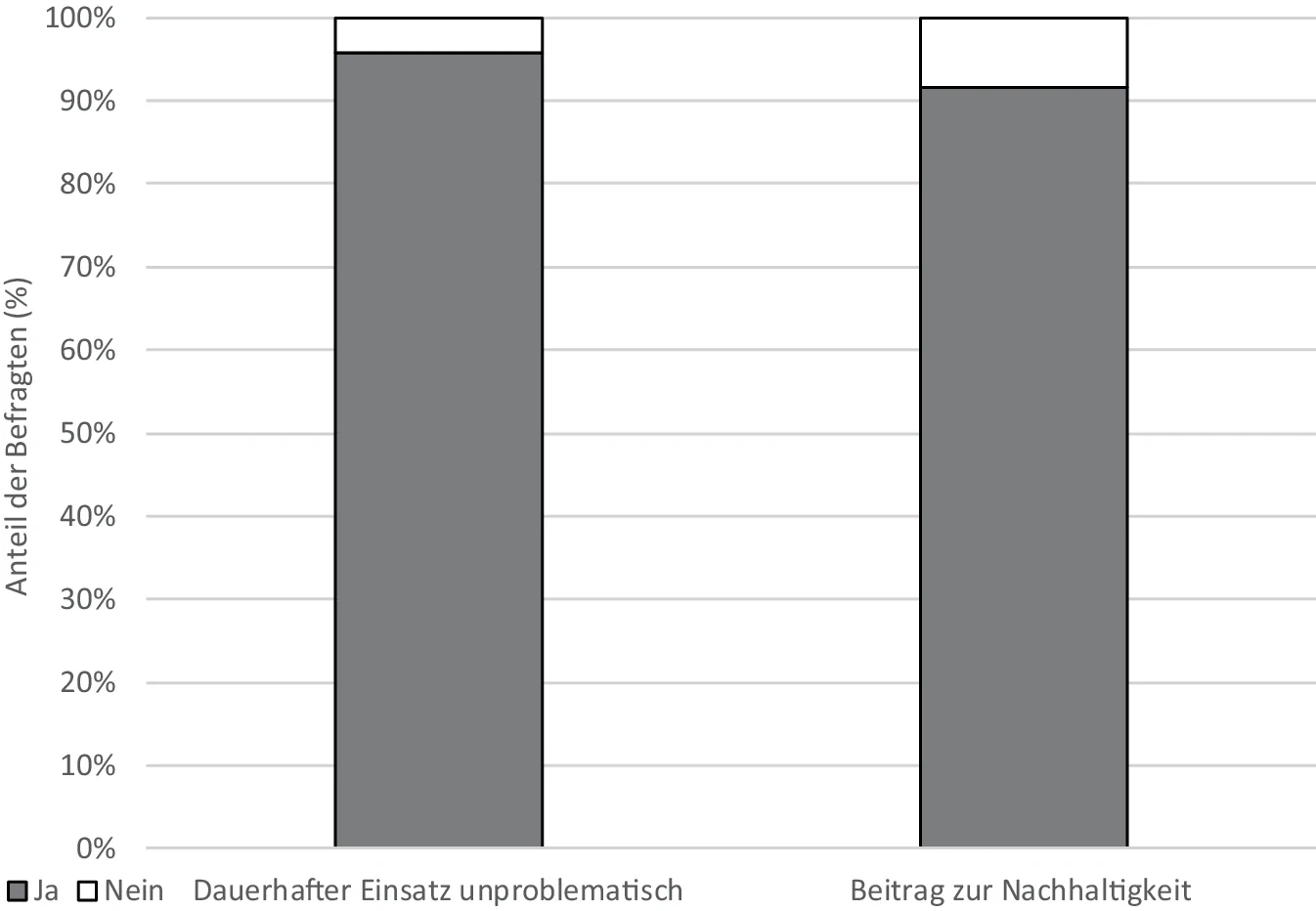
Befragung des Personals zu Mehrwegtextilien: 96 % der Befragten schätzten einen dauerhaften Einsatz von Mehrwegtextilien als unproblematisch ein, und 92 % sahen in der Nutzung von Mehrwegtextilien einen Beitrag zur Nachhaltigkeit in Operationssälen

Insgesamt wurden 24 Fragebögen durch das Personal ausgefüllt (46 % OP-Funktionsdienst, 54 % ärztlicher Dienst). 33 % des befragten Personals waren unter 30 Jahre alt. Die Berufserfahrung betrug im Median 5 bis 10 Jahre. Bei Befragung des Stellenwertes der Nachhaltigkeit wurde von 96 % bzw. 92 % des befragten Personals angegeben, dass Nachhaltigkeit im Alltag bzw. im OP als wichtig eingestuft wird. 83 % der Befragten schätzten Mehrwegtextilien in Bezug auf hygienische Aspekte und 79 % in Bezug auf den Materialkomfort vorteilhaft gegenüber Einwegtextilien ein. Auf einer Skala von 1 (unzufrieden) bis 5 (sehr zufrieden) wurden Materialkomfort von Mehrwegtextilien im Mittel mit 3,25 (± 1,18), Handhabung mit 3,04 (± 1,23) Punkten und Hygiene mit 3,63 (± 0,77) Punkten bewertet. 96 % der Befragten schätzten die Etablierung von Mehrwegtextilien im OP insgesamt als unproblematisch ein, auch wenn 30 % noch Verbesserungspotential sahen. Insgesamt empfanden 92 % des befragten Personals die Nutzung von Mehrwegtextilien als relevanten Beitrag zur Nachhaltigkeit (Abb. 2).

Die Berufserfahrung korrelierte signifikant mit dem Alter (ρ = 0,92; *p* < 0,001), jedoch nicht mit der Bewertung der Mehrwegtextilien oder dem subjektiven Stellenwert der Nachhaltigkeit. Mitarbeitende des OP-Funktionsdienstes gaben signifikant mehr Optimierungsbedarf im Hinblick auf den Komfort an (x^2^(1) = 6,33; *p* = 0,023). Es zeigte sich eine positive signifikante Korrelation zwischen subjektivem Stellenwert der Nachhaltigkeit und hygienischem Sicherheitsgefühl (ρ = 0,52; *p* = 0,009). Dazu schätzten Befragte, die mit dem Materialkomfort zufriedener waren, den Beitrag von Mehrwegtextilien zur Nachhaltigkeit signifikant höher ein (ρ = 0,43; *p* = 0,035). Befragte, die den Materialkomfort und die Handhabung besser bewertet hatten, zeigten signifikant höhere Zustimmung zur dauerhaften Etablierung von Mehrwegmaterialien (ρ = 0,70; *p* < 0,001 bzw. ρ = 0,71; *p* < 0,001).

## Diskussion

In dieser Studie konnte gezeigt werden, dass die Nutzung von Mehrwegabdeckungen im OP zu einer Reduktion des krankenhausspezifischen Abfalls um bis zu 40 % des Volumens bzw. 34 % des Gewichtes führen kann, ohne Nachteile auf Vor- und Nachbereitungszeiten sowie OP-Dauer zu haben, was die Studienhypothese bestätigt.

## Abfallmanagement

Das Abfallmanagement in Krankenhäusern spielt neben Energie- und Wasserhaushalt eine entscheidende Rolle bei der Entwicklung zu einem nachhaltigen Gesundheitssystem. Besonders in chirurgischen Fachbereichen, die in Operations- und Eingriffsräumen ca. 30 % der Abfälle und einen hohen Anteil der CO_2_-Emissionen im Krankenhaus produzieren, können durch gezielte Maßnahmen zur Verbesserung der Klimabilanz beitragen [7, 10]. Es ist bereits bekannt, dass eine effektive Wertstofftrennung und Einschleusung von recyclebaren Wertstoffen in den Rohstoffkreislauf eine signifikante Reduktion der CO_2_-intensiven krankenhausspezifischen Abfälle bewirken kann [9, 14]. Krankenhausspezifische Abfälle müssen aufgrund der potentiellen Kontamination unter hohen Temperaturen > 1100 °C verbrannt werden, was eine CO_2_-Emission von ca. 0,95–3 kg CO_2_/kg Abfälle ausstößt [2].

## Mehrwegtextilien

Während die Nutzung von Mehrweginstrumenten mittlerweile gut untersucht und in vielen Kliniken als Standard etabliert wurde [8, 15], scheinen Einwegmaterialien weiterhin ca. 30 % der Abfallmenge in OP auszumachen [16]. Einen relevanten Anteil dieser Einwegmaterialien stellen Textilien dar, die aufgrund der Kontamination mit Körperflüssigkeiten und Gewebe als krankenhausspezifische Abfälle eingestuft werden [17]. Durch Nutzung von Mehrwegbettbezügen sowie Mehrweghauben konnten im Vergleich zu Einwegmaterialien bereits deutliche Einsparungen der CO_2_-Emissionen nachgewiesen werden [18, 19]. Auch Mehrwegkittel haben sich bereits in mehreren Studien durch eine signifikante Reduktion der Abfälle und eine bessere Ökobilanz ausgezeichnet [11]. Außerdem zeigen Mehrwegtextilien gegenüber Einwegtextilien signifikant bessere Ergebnisse in Studien zu Sterilität und Sicherheit sowie Haltbarkeit der Materialien [11, 20]. Zum direkten ökologischen Vergleich zwischen Einweg- und Mehrwegabdeckungen gibt es dagegen bisher nur wenig Literatur. Aufgrund der bereits regulär genutzten Mehrwegkittel konnten in dieser Studie Mehrwegabdeckungen isoliert betrachtet werden. Insgesamt wurden dadurch im Mittel 1,82 kg pro Operation sowie in 10 Tagen mehr als 300 kg krankenhausspezifischer Abfall eingespart. Der signifikante Anstieg des Gewichtes bei gleichbleibendem Volumen des potentiell recyclebaren Wertstoffes könnte dabei auf die festeren Verpackungen der Mehrwegtextilien zurückzuführen sein, die in dieser Studie aufgrund des geringen Eigengewichtes der Einzelverpackungen nicht separat erfasst wurden.

Der krankenhausspezifische Abfall unter Nutzung von Einwegabdeckungen zeigt eine große Standardabweichung für das gemessene Volumen, nicht aber für das Gewicht. Dies ist im Wesentlichen auf das heterogene orthopädisch-unfallchirurgische Eingriffsspektrum mit unterschiedlichen Abdeckungsgrößen zurückzuführen, die sich mehr im Volumen, weniger aber im Gewicht abbilden.

Die gemessene Gesamtabfallmenge von 6,3 kg pro Operation sowie der Anteil an potentiell recyclebaren Wertstoffen entsprechen bisherigen, stark von den beobachteten Operationen abhängigen, Studien [21, 22]. Diese Studie zeichnet sich dahingehend aufgrund des breiten gemischt orthopädisch-unfallchirurgischen Operationsspektrums eines Maximalversorgers und Level-I-Traumazentrums und eine resultierende hohe Übertragbarkeit aus.

## CO_2_-Emissionen

Die Berechnung des ökologischen Fußabdruckes von Materialien stellt aufgrund der verschiedenen Komponenten und unterschiedlichen beteiligten Dienstleister eine große Herausforderung dar [23]. Beim Vergleich der CO_2_-Emissionen von Einweg- und Mehrwegabdeckungen können primär die klinikunabhängigen Faktoren Produktion, Waschen/Sterilisation sowie Entsorgung bei fest definierten Abdeckungs-Sets betrachtet werden. Die Produktionsemissionen wurden in Höhe von 7 kg CO_2_/Einweg- und 32,5 kg CO_2_/Mehrweg-Set angegeben [24]. Bei Entsorgung entstehen, je nach Quelle, ca. 1– 3 kg CO_2_/kg krankenhausspezifischem Abfall, was bei 1,135 kg schweren Einweg-Sets ca. 1,135–3,405 kg CO_2_ und bei 3,026 kg schweren Mehrweg-Sets ca. 3,026–9,078 kg CO_2_ entspricht [2, 24, 25]. Diese Werte werden anhand der Anwendungsrate der Mehrwegabdeckungen angepasst, die in dieser Studie übereinstimmend mit anderen Beobachtungen im Mittel 65 betrug [12, 24]. Für Waschen und Sterilisation wurde bei Einwegartikeln einmalig 0,1 kg CO_2_ und bei Mehrwegmaterialien aufgrund des höheren Gewichtes 1,7 kg/Nutzung angegeben [24]. Insgesamt könnten damit durch Nutzung von Mehrwegartikeln 6,0–8,2 kg CO_2_/Anwendung bzw. 389,2–530,7 kg CO_2_/genutztem Abdeckungs-Set in Bezug auf Produktion, Wiederverwertung und Entsorgung eingespart werden. Neben diesen Faktoren ist ein Großteil der CO_2_-Emissionen auf Transportwege zurückzuführen [23]. Bei Produktionsstandorten sowie gleicher Entfernungen zu Wäscherei wie Entsorgung könnten diese Faktoren im direkten Vergleich vernachlässigt werden. Ansonsten sollten diese Transportwege sowie die variierenden Produktionsprozesse bei der Berechnung individuell nach Klinikstandort, Transportdienstleister und Hersteller ermittelt und in die Abwägung zwischen Nutzung von Einweg- und Mehrwegmaterialien einbezogen werden.

## Zufriedenheit

Insgesamt gaben 96 % des befragten Personals aus ärztlichem oder OP-Funktionsdienst an, eine langfristige Einführung von Mehrwegabdeckungen als unproblematisch einzuschätzen, und 92 % sahen in der Nutzung von Mehrwegtextilien einen Beitrag zur Nachhaltigkeit. Unsere Hypothese bestätigend erfuhr die Etablierung von Mehrwegtextilien eine hohe Akzeptanz durch das Personal und wurde nicht nachteilig gegenüber Einwegmaterialien empfunden, auch wenn noch Optimierungsbedarf besteht. Dies passt auch zu den positiven Bewertungen von Mehrwegtextilien in anderen Studien [26, 27]. Die Zustimmung zur dauerhaften Etablierung war signifikant von empfundenem Materialkomfort und Handhabung abhängig. Dies zeigt, dass diese Faktoren für die Akzeptanz neuer Materialien und Umstrukturierungen essenziell sind. Befragte aus dem OP-Funktionsdienst gaben einen höheren Optimierungsbedarf des Komforts insbesondere von Gewicht und Größen an, was auf das deutlich höhere Gewicht der Mehrwegmaterialien, die vom OP-Funktionsdienst transportiert werden, zurückzuführen sein könnte.

Die signifikante Korrelation zwischen dem subjektiven Stellenwert der Nachhaltigkeit im OP und der hygienischen Sicherheit sowie der Anteil von 17 % der Befragten mit Bedenken dahingehend ist erstaunlich, da die Nutzung von Mehrwegkitteln in der Klinik bereits zuvor etabliert war. Unklar bleibt, ob Befürchtungen von hygienischen Mängeln die subjektive Bedeutung der Nachhaltigkeit bei den Befragten schmälern, oder ob Interesse an Nachhaltigkeit im OP zu einem größeren Wissen auf diesem Gebiet und damit Aufklärung von Bedenken führt. Vorbehalte in Bezug auf die hygienische Sicherheit von Mehrwegmaterialien scheint trotz vieler Studien noch immer ein relevantes Thema zu sein [10], obwohl eine bessere Haltbarkeit, Beständigkeit und Sterilität bei Mehrwegtextilien im Vergleich zu Einwegtextilien in vielen Studien bewiesen wurde [11, 20]. Es ist also von großer Bedeutung, weitere Schulungen durchzuführen, um solche Bedenken aufzuklären.

Positiv ist zu werten, dass die Zufriedenheit, der Stellenwert der Nachhaltigkeit und die Einschätzung des Beitrags von Mehrwegtextilien zur Nachhaltigkeit keine Differenzen in Bezug auf Alter oder Berufserfahrung zeigten, sondern alle Befragten so gleichermaßen interessiert und offen für die Evaluation von Mehrwegtextilien waren, auch wenn ein Bias aufgrund der anonymisierten Befragung diskutiert werden kann. Dies könnte auch auf die Vertrautheit durch regulär genutzte Mehrwegkittel und die zuvor durchgeführte interprofessionelle Schulung begünstigt sein. Passend zu dieser Studie werden auch in anderen Publikationen ein steigendes Interesse sowie Bewusstsein für Nachhaltigkeit bei einem Großteil des medizinischen Personals beschrieben, und oft verhindert Unwissenheit über Maßnahmen wie Recycling und Müllreduktion die Umsetzung [28]. Daneben stellt auch eine mangelnde Studienlage oft ein Hindernis dar [29]. Studien wie diese können mit Hilfe wissenschaftlicher Fakten den Grundstein für eine langfristige Umstrukturierung legen, teilnehmendes Personal langfristig sensibilisieren und aktiv einbinden, um Handhabung und Komfort und damit die Akzeptanz noch weiter zu verbessern [30].

## Schlussfolgerung

Die Nutzung von Mehrwegabdeckungen kann den anfallenden Abfall im Operationssaal signifikant reduzieren und damit zu einer effektiven Reduktion der CO_2_-Emissionen beitragen, ohne die Abläufe der Operationen relevant zu beeinflussen. Allerdings müssen auch Emissionen durch Transportwege und Produktion individuell nach Klinik und Hersteller berücksichtigt werden. Die Nutzung von Mehrwegtextilien erfuhr eine hohe Akzeptanz durch das Personal, und die dauerhafte Etablierung wurde als unproblematisch und als wertvoller Beitrag zur Nachhaltigkeit angesehen. Eine langfristige Implementierung von Mehrwegtextilien stellt damit eine wertvolle Möglichkeit auf dem Weg zu einem nachhaltigeren Gesundheitssystem dar.

## Fazit für die Praxis

Die Nutzung von Mehrwegtextilien kann zu einer signifikanten Reduktion des anfallenden Abfalls im Operationssaal beitragen. Daraus kann auch eine Reduktion der CO_2_-Emissionen resultieren, allerdings müssen auch Emissionen durch Transportwege und Produktion individuell nach Klinik und Hersteller berücksichtigt werden. Die Operationsabläufe sowie die Operationszeiten wurden durch die Umstellung auf Mehrwegabdeckungen nicht relevant beeinflusst. Die Nutzung von Mehrwegtextilien erfuhr eine hohe Akzeptanz durch das Personal. Schulungen und Fortbildungen sind für eine problemlose dauerhafte Etablierung essenziell. Eine langfristige Implementierung von Mehrwegtextilien stellt eine wertvolle Möglichkeit auf dem Weg zu einem nachhaltigeren Gesundheitssystem dar.

## Data Availability

Die im Rahmen dieser Studie generierten Daten sind aus datenschutzrechtlichen Gründen nicht veröffentlicht, können jedoch auf begründete Anfrage beim korrespondierenden Autor zur Verfügung gestellt werden.

## References

[1] Eckelman MJ, Huang K, Lagasse R, Senay E, Dubrow R, Sherman JD (2020) Health care pollution and public health damage in the united states: An update. Health Aff 39:2071–2079 10.1377/hlthaff.2020.01247 33284703

[2] Richter H, Pecher S (2023) Abfallvermeidung und -entsorgung im OP. OP-Journal 39:35–48

[3] Eckelman MJ, Sherman JD (2018) Estimated Global Disease Burden From US Health Care Sector Greenhouse Gas Emissions. Am J Public Health 108(S2):S120–S122 10.2105/AJPH.2017.303846 PMC5922190 29072942

[4] Trojan A, Fehr R (2020) Sustainable urban health: conceptual foundations and current initiatives. Bundesgesundheitsblatt - Gesundheitsforsch - Gesundheitsschutz 63:953–961 10.1007/s00103-020-03187-8 PMC8219555 32643005

[5] Wang H, Horton R (2015) Tackling climate change: The greatest opportunity for global health. Lancet 386:1798–1799 10.1016/S0140-6736(15)60931-X 26111438

[6] Lenzen-Schulte M (2019) Wenn Abfall nicht einfach Müll ist. Dtsch Arztebl Int 116:A96–A97

[7] McAleese T, Jagiella-Lodise O, Roopnarinesingh R, Cleary M, Rowan F (2024) Sustainable orthopaedic surgery: Initiatives to improve our environmental, social and economic impact. Surgeon 22(4):215–220 10.1016/j.surge.2023.06.005 37455209

[8] Pradere B, Mallet R, de La Taille A, Bladou F, Prunet D, Beurrier S et al (2023) Climate-smart Actions in the Operating Theatre for Improving Sustainability Practices: A Systematic Review. Eur Urol 83:331–342 10.1016/j.eururo.2022.01.027 35151515

[9] Mastronardi M, Bortul M, Dragani F, Biloslavo A, de Manzini N, Palmisano S (2025) Implementing recycling in the operating room: a single-center experience. Surg Endosc 39:1323–1332 10.1007/s00464-024-11502-6 39762609

[10] Lattanzio S, Stefanizzi P, D’ambrosio M, et al (2022) Waste Management and the Perspective of a Green Hospital-A Systematic Narrative Review. Int J Environ Res Public Health 19(23):15812 10.3390/ijerph192315812 PMC9738387 36497884

[11] Egetemeyer J, Jacob S, Lehmann F, Guba M, Werner J, Börner N (2023) Nachhaltigkeit im OP – korrelieren Sterilität, Sicherheit und Service auch mit einer ressourcenschonenden Verwendung von Medizinprodukten? Zentralblatt für Chir - Zeitschrift für Allg Visz Thorax- und Gefäßchirurgie 148:120–123 10.1055/a-2045-0078 37015246

[12] von Eiff W (2016) Klinische Textilien im OP der Zukunft: Wirtschaftlichkeit und Qualität von Mehrweg-Produkten. Das Krankenhaus 777–780.

[13] Cherif C, Günther E, Jatzwauck L, Mecheels S (2009) Evaluierung von OP-Textilien. Ergebnisse einer Untersuchung nach hygienischen, ökonomischen und ökologischen Gesichtspunkten. Dresden

[14] Evliya Felek BN, Karadağ Erkoç S, Özçelik M, Yörükoğlu D (2025) Impact of waste segregation training on medical and recyclable waste in an operating theater a quasi experimental study. Sci Rep 15:1–10 10.1038/s41598-025-02797-z PMC12106621 40419647

[15] Desclée de Maredsous R, Huet S, Brichoux S, Charissoux JL, Marcheix PS (2022) User satisfaction with single-use instruments versus reusable instruments for treating distal radius fractures with locking volar plates. Orthop Traumatol Surg Res 108(5):103217 10.1016/j.otsr.2022.103217 35093567

[16] Rotaru J, Miller P, McGarry L, O’Byrne JM (2025) Environmental impact of single-use items and their contamination levels in elective orthopaedic surgery. Surgeon 23:296–300. 10.1016/j.surge.2025.07.007 40803931

[17] Wyssusek KH, Keys MT, van Zundert AAJ (2019) Operating room greening initiatives – the old, the new, and the way forward: A narrative review. Waste Manag Res 37:3–19 10.1177/0734242X18793937 30132405

[18] Donahue LM, Petit HJ, Thiel CL, Sullivan GA, Gulack BC, Shah AN (2024) A Life Cycle Assessment of Reusable and Disposable Surgical Caps. J Surg Res 299:112–119 10.1016/j.jss.2024.04.007 38749314

[19] Chang JH, Woo KP, Silva de Souza Lima Cano N, Bilec MM, Camhi M, Melnyk AI et al (2024) Does reusable mean green? Comparison of the environmental impact of reusable operating room bed covers and lift sheets versus single-use. Surgeon 22:236–241 10.1016/j.surge.2024.05.003 38862376

[20] Collard MK, Vaz A, Irving H, Khan MF, Mullis D, Brady D et al (2023) Reusable cloth masks in operating theatre. Br J Surg 110:1260–1263 10.1093/bjs/znad104 37119205

[21] Kooner S, Hewison C, Sridharan S, Lui J, Matthewson G, Johal H et al (2020) Waste and recycling among orthopedic subspecialties. Can J Surg 63:E278–E283 10.1503/cjs.018018 PMC7828994 32437094

[22] Pegg M, Rawson R, Okere U (2022) Operating room waste management: A case study of primary hip operations at a leading National Health Service hospital in the United Kingdom. J Heal Serv Res Policy 27:255–260 10.1177/13558196221094488 35635489

[23] Rizan C, Steinbach I, Nicholson R, Lillywhite R, Reed M, Bhutta MF (2020) The Carbon Footprint of Surgical Operations: A Systematic Review. Ann Surg 272:986–995 10.1097/SLA.0000000000003951 32516230

[24] https://www.sitex.de/aktuelle-lca-op-mehrweg-vs-einweg-textilien/ (Zugegriffen am 31. März 2026)

[25] Southorn T, Norrish AR, Gardner K, Baxandall R (2013) Reducing the carbon footprint of the operating theatre: A multicentre quality improvement report. J Perioper Pract 23:144–146 10.1177/175045891302300605 23909168

[26] van Nieuwenhuizen KE, Friedericy HJ, van der Linden S, Jansen FW, van der Eijk AC (2024) User experience of wearing comfort of reusable versus disposable surgical gowns and environmental perspectives: A cross-sectional survey. BJOG An Int J Obstet Gynaecol 131:709–715 10.1111/1471-0528.17685 37806784

[27] Agarwal D, Bharani T, Armand W, Slutzman JE, Mullen JT (2023) Reusable scrub caps are cost-effective and help reduce the climate footprint of surgery. Langenbeck’s Arch Surg 408:1–5 10.1007/s00423-023-03107-9 37707671

[28] Patel SD, Smith-Steinert R (2021) Greening the operating room, one procedure at a time. J Clim Chang Heal 2:1–5

[29] Balch JA, Krebs JR, Filiberto AC, Montgomery WG, Berkow LC, Upchurch GR et al (2023) Methods and evaluation metrics for reducing material waste in the operating room: a scoping review. Surg (United States) 174:252–258 10.1016/j.surg.2023.04.051 PMC12290801 37277308

[30] Carmona-Pomada B, Diaz-Co L, Azaroual El Bachiri H, Nieto-Lorente N, Muriel-Serrano G, Zarza-Sánchez L, et al. Segregation and Recycling in the Operating Room. An Intervention to Accelerate the Decarbonisation Process in the Health Sector. J Clin Nurs. 2025;1–12. 10.1111/jocn.17731 PMC12489432 40084717

